# Effect of dental intervention on improvements in metabolic syndrome patients: a randomized controlled clinical trial

**DOI:** 10.1186/s12903-020-01373-3

**Published:** 2021-01-06

**Authors:** Midori Doke, Yuriko Komagamine, Manabu Kanazawa, Maiko Iwaki, Hiroyuki Suzuki, Yasunari Miyazaki, Tetsuya Mizuno, Kaori Okayasu, Shunsuke Minakuchi

**Affiliations:** 1 Gerodontology and Oral Rehabilitation, Graduate School of Medical and Dental Sciences, Tokyo Medical and Dental University, Yushima, Bunkyo-ku, Tokyo, Japan; 2 General Dentistry, Graduate School of Medical and Dental Sciences, Tokyo Medical and Dental University, Tokyo, Japan; 3 Department of Respiratory Medicine, Graduate School of Medical and Dental Sciences, Tokyo Medical and Dental University, Tokyo, Japan; 4 Department of Health Science and Physical Education, College of Liberal Arts and Sciences, Tokyo Medical and Dental University, Tokyo, Japan; 5 Yokohama City Minato Red Cross Hospital Medical Center for Allergic and Immune Diseases, Yokohama, Japan

**Keywords:** Metabolic syndrome, Obesity, Dental intervention, Periodontal treatment, Prosthodontic treatment

## Abstract

**Background:**

Metabolic syndrome (MetS), caused by the accumulation of visceral fat, is considered a major cause of cardiovascular disease. This randomized controlled trial aimed to clarify the effect of dental intervention, including prosthodontics and/or periodontal treatment, combined with dietary and exercise guidance on MetS.

**Methods:**

In total, 112 patients who met the Japanese waist circumference criteria of MetS were recruited. The intervention group (ITG) received dental intervention along with dietary and exercise guidance, while the control group (CTG) received dietary and exercise guidance alone. Three outcome measurements were obtained before intervention (BL), 1 month after intervention (1M), and 3 months after intervention (3M).

**Results:**

Body water rate (*p* = 0.043) was significantly higher in ITG than in CTG at 1M. Simultaneously, fasting blood sugar level (*p* = 0.098) tended to be lower in ITG than in CTG. Lean mass (*p* = 0.037) and muscle mass (*p* = 0.035) were significantly higher and body weight (*p* = 0.044) significantly lower in ITG than in CTG at 3M. Body mass index (*p* = 0.052) tended to be lower in ITG than in CTG.

**Conclusions:**

Dental intervention combined with lifestyle guidance may improve anthropometric status and reduce the risk of MetS.

***Trial registration*:**

University Hospital Medical Information Network Center Unique UMIN000022753. https://upload.umin.ac.jp/cgi-open-bin/ctr/ctr_view.cgi?recptno=R000026176.

## Background

According to the World Health Report of the World Health Organization [1] (WHO), metabolic syndrome (MetS) has been identified as the main cause of cardiovascular disease, which has been increasing worldwide. The prevalence of MetS among Japanese nationals aged 40–74 years has been estimated at 19,400,000 (15.3%) by the Japan Ministry of Health, Labor and Welfare [2].

MetS has obesity, diabetes, hypertension, dyslipidemia, and insulin resistance (IR) at its base, and is caused by the accumulation of visceral fat [3]. This IR is characterized by an impaired insulin function, and lack of exercise and abdominal visceral fat have been indicated in IR. Japanese criteria of MetS [4] have been based on the National Cholesterol Education Program–the third revision of the Adult Treatment Panel (NCEP ATP III) [5]. MetS was defined as a status with abdominal obesity (men: ≥ 85 cm, women: ≥ 90 cm circumference at the navel), in addition to more than two of the following three statuses: diabetes (fasting blood sugar level: FBS level ≥ 110 mg/dl), hypertension (blood pressure ≥ 130/85 mmHg), dyslipidemia (triglyceride level: TG level ≥ 150 mg/dl and/or high density lipo-protein level: HDL level ≤ 40 mg/dl).

NCEP ATP III stated two treatment goals in MetS [5]. The first goal was to improve on the major causes of MetS, such as obesity and lack of exercise, and the second goal was the improvement of MetS-related factors, which include diabetes, hypertension, and dyslipidemia. To reduce the risk of MetS, weight reduction and increased physical exercise, maintained concurrently with diet therapy, would be necessary. Thromboprophylaxis medication, combined with general treatment for diabetes, hypertension, and dyslipidemia, should be administered to treat MetS-related factors.

The relationship between MetS and oral environment has been reported. Obese people tend to have a higher prevalence rate of periodontitis than do people with normal stature [6]. Al-Zahrani reported the significant relation between body fat and periodontal disease [7]. The mechanism of pathological correlation between MetS and periodontitis has been explained by Lamster and Pagan, who had reported that inflammatory cytokines mediate host response to periodontal pathogens by activating leucocytes [8]. These cytokines are mediated by adipocytokines secreted from abdominal visceral fat. Therefore, abdominal obesity has been considered to have a strong relationship with periodontitis [9]. This cascade would also increase IR, which would induce systemic imbalance in glucose and lipid metabolism, leading to hyperglycemia and dyslipidemia [10]. In a state of diabetic dyslipidemia, insulin resistance is strongly correlated with elevated TG levels, which would increase serum HDL and LDL, and cause abnormalities in glucose metabolism [11]. Hyperglycemia would cause hyper-inflammatory response to periodontium, and impairs resolution of inflammation and repairmen of tissue [12]. From these reports, serum TG, HDL, LDL, glucose and HbA1c have been related to status of both MetS and Periodontitis. As a behavioral fact, Kobayashi suggested in their longitudinal study that the frequency of tooth brushing was related to lower prevalence of MetS [13]. However, those studies indicate correlation between MetS and periodontitis, but the causal relation remains unclear. Considering these reports, obese people tend to be neglectful of oral health behaviors, and this seems to be one of the factors in development of periodontitis, but the impact of oral hygiene on MetS still remains unclear.

Some clinical trials performed periodontal treatment on participants with diabetes [14, 15], obesity [16], and MetS [17], which revealed that inflammatory markers and glycated hemoglobin (HbA1c) levels were reduced after the periodontal therapy. Saengtipbovorn and Taneepanichskul conducted a clinical trial that combined lifestyle guidance and dental care in participants with diabetes [18]. Apart from other previous reports in which they performed periodontal treatment only as intervention, this trial has its novelty in combining lifestyle therapy with periodontal treatment. However, no clinical trial has investigated the combined effect of lifestyle guidance and dental intervention on MetS. By combining dental intervention with lifestyle therapy, additional improvement on MetS would be expected.

Therefore, this randomized controlled trial (RCT) aimed to clarify the effect of dental intervention, including prosthodontics and/or periodontal treatment, combined with dietary and exercise guidance on MetS. The null hypothesis is that no difference exists between the waist circumference and anthropometric measurements of the intervention group (ITG) that received dental intervention along with dietary and exercise guidance and the control group (CTG) that received dietary and exercise guidance alone.

## Materials and methods

This study followed the 2010 Consolidated Standards for Reporting Trials (CONSORT) statement. The trial protocol was approved by the Ethics Committee of the Faculty of Dentistry, Tokyo Medical and Dental University (TMDU; Registration No. D2016-028, date of final registration 25/1/2019), and is registered in the University Hospital Medical Information Network Center (UMIN-CTR Clinical Trial, Unique Trial No. UMIN000022753). All participants provided written informed consent before participation in the study. We recruited 112 patients who met the Japanese waist circumference criteria of MetS. This study was conducted in TMDU Dental Hospital. Participants consisted of TMDU staff and patients who visited TMDU Medical Hospital and/or Dental Hospital. Inclusion criteria were as follows: age ≥ 40 years, at the time when the consent form was signed; waist circumference criteria for MetS of ≥ 85 cm for men and ≥ 90 cm for women at the navel; missing tooth according to Eichner’s criteria A2, A3, B1, B2, B3, B4, C1, C2, C3, and/or patients with light–moderate periodontitis (4 mm ≤ PD < 6 mm); and signing of consent form after the explanation and understanding of the study objectives. In this study, we have modified the criteria of CPI, and defined PD ≥ 4 mm as mild to moderate periodontitis, where the patients would need professional dental care [19]. We excluded PD > 6 mm, which is a status that needs periodontal surgery, since the long healing period after surgery might exceed the ethical time limit of study period. In the Eichner’s classification, patients would be divided into three main groups as Eichner’s index (A, B, and C), according to the occlusal status supported by remaining teeth [20]. Occlusion is supported by four occlusal areas in the healthy dentition composed of the right and left premolars and molars, and areas that support occlusion are called occlusal support areas. Eichner classification groups A, B, and C were as follows: (A) Occlusal contacts are present in all four posterior support zones; (B) occlusal contacts are present in 1–3 zone(s); (C) occlusal contact is in the anterior region only and / or no occlusal contacts.

Patients were excluded from the study as per the following criteria: involvement in regular dietary and/or exercise guidance outside this study, barriers to attend and/or achieve dietary and exercise guidance and the study objectives, changes in medication prescription within the past 3 months, severe cardiovascular disease, and hyper/hypo function of the thyroid. Further, patients with severe disorders of the liver and/or kidneys, undergoing treatment for cancer, with pregnancy, or with possibility of pregnancy, and others who were judged as ineligible for this study by the principal investigator were excluded from the study. Participants were recruited using the following methods: all the TMDU employees who participated in regular medical examinations received recruiting brochures for this study, with a clear statement that their participation is optional and not compulsory or included as part of their business; posters and flyers were advertised in TMDU Medical and Dental Hospital; and participant selection was entrusted and contracted to competent companies.

A double-blinded parallel group RCT was initiated inside a single facility (TMDU Dental Hospital). Participants were allocated to ITG and CTG. All the patients who had gone through a baseline examination received dietary and exercise guidance, which consisted of the Total Fitness Analysis System (TFAS) [21] and a video-programmed dietary and exercise lecture. TFAS is a web application available on the Internet of TMDU, which was created as a life management tool by the TMDU Department of Health Science and Physical Education. This application has its concept based on “Health Promotion 2006: Physical Activity, Exercise, and Physical Fitness” [22], “Exercise Guide 2006” [23], and “Japanese Food Guide Spinning Top” [24]. Participants typed in their recent dietary and exercise records, and they received their results and feedback from TFAS. Thereafter, they were instructed to watch 60 min of the video lecture about diet and exercise. This program was directed by a specialist in health science. There was no enforcement of specific diet or exercise since this guidance was intended to enhance motivation to improve health. The initial session of Specific Health Guidance [25] took a maximum of 80 min; therefore, the intensity of our trial seemed to be equivalent.

ITG underwent non-surgical periodontal treatment and/or prosthodontics, while CTG would receive their dental intervention after the study period. Three months was considered as the ethical limit to keep the controls away from undergoing needed dental treatment. Non-surgical periodontal treatment included tooth-brushing instruction (TBI) and supra/subgingival scaling and root planing (SRP). Prosthodontic treatment was performed for patients with missing tooth/teeth according to Eichner’s classification: A2, A3, B1, B2, B3, B4, C1, C2, and C3. Fixed partial dentures and removable partial dentures were used to fix the defects. For patients who needed both interventions, periodontal treatment was performed first, and then the prosthodontics was performed next. Six dentists with > 6 years of clinical experience had performed the dental intervention as operators. As a coordinator, a dentist who was not involved in dental intervention for participants, performed randomization and dietary and exercise guidance, outcome assessment, and statistical analysis.

### Outcomes

The three following outcome measurements were obtained: first, before intervention (BL); second, 1 month after intervention (1M); and third, 3 months after intervention (3M). To avoid missing data, the investigator re-checked the questionnaires after participants had filled them. The primary outcome of this study was focused on waist circumference, which is the simplest method to assess visceral fat accumulation with accuracy [26]. Waist circumference was measured horizontally around the navel in an upright standing posture. Along with the primary outcome, secondary outcomes, such as blood pressure, blood sample test, and anthropometric measurements using body composition analyzer, were assessed.

Blood pressure was measured to determine the presence of hypertension. The patient’s arm was stretched and positioned at the level of the heart in an upright sitting position. Thereafter, the cuff band was wrapped around their arm at the brachial artery. Subsequently, the blood pressure was measured using an electronic sphygmomanometer (OMRON digital automatic sphygmomanometer HEM-1000) [27]. Blood samples were taken to determine hyperglycemia and dyslipidemia through the following indices: TG, HDL, low-density lipoprotein (LDL), FBS, and HbA1c levels. Further, 10 ml blood samples, taken from each patient at the cubital fossa, were then centrifuged and analyzed. Patients were asked to fast for at least 6 h before the tests.

TANITA MC-780A [28], a multi-frequency body composition analyzer, was used for the anthropometric measurements. Weight, BMI, body fat percentage, body fat mass, lean body mass, body muscle mass, estimated bone mass, body water mass, and body water percentage were scanned and assessed.

The number of teeth present was counted. Thereafter, the following dental measurements were performed to assess the periodontal statuses of patients: pocket depth (PD) and bleeding on probing (BOP), measured at six sites per tooth.

The sample size estimation was calculated from the change in waist circumference based on an RCT conducted by Munakata [29]. A difference in waist circumference > 3.0 cm (standard deviation: 5.0 cm) within the two groups was regarded as statistically significant. Following the normal distribution of waist circumference, 80 participants were needed to obtain a significance level of 0.05 along with 95% confidence interval. Considering the dropout rate of 20%, 100 participants (50 in each group) were determined to be the goal of recruitment. For the randomization, sealed opaque envelope system was used to randomly allocate participants into ITG and CTG. For each patient, the coordinator had picked up sealed opaque envelope, and then the treatment allocation was generated. The allocation was written on a paper, which was folded with silver paper, and then was sealed into an opaque envelope. Coordinator had opened the envelope after writing the patient’s name onto the envelope. Also, the coordinator had done the preparation of envelopes and enrollment of participants and the assignment of participants. This trial was carried out as double-blinded parallel group RCT, since both the participants and the operator had been unaware of the allocation. Participants were blinded from the group allocation because they were not informed about the groups to which they belonged nor their study schedule. Operators were blinded because the coordinator scheduled the treatment, and they were unaware of the allocation. However, the blinding of the coordinator who performed both the allocation and assessment had been impossible.

### Statistical methods

Student-T test was applied for assessment between groups at baseline, and comparison between dropouts and those who retained. One-way analysis of covariance (ANCOVA) was applied to analyze the difference between the two groups in each assessment of the primary and the secondary outcomes. The difference between each assessment within groups was analyzed using repeated analysis of variance (Repeated ANOVA). Subgroup analysis for patients with missing teeth and those with periodontitis alone was performed to assess the deviation from treatment types using ANCOVA. Analyses were performed using SPSS Version17 software (IBM Japan) [30]. Significance level of 0.05 has been used in each analysis.

## Results

**Fig. 1:**
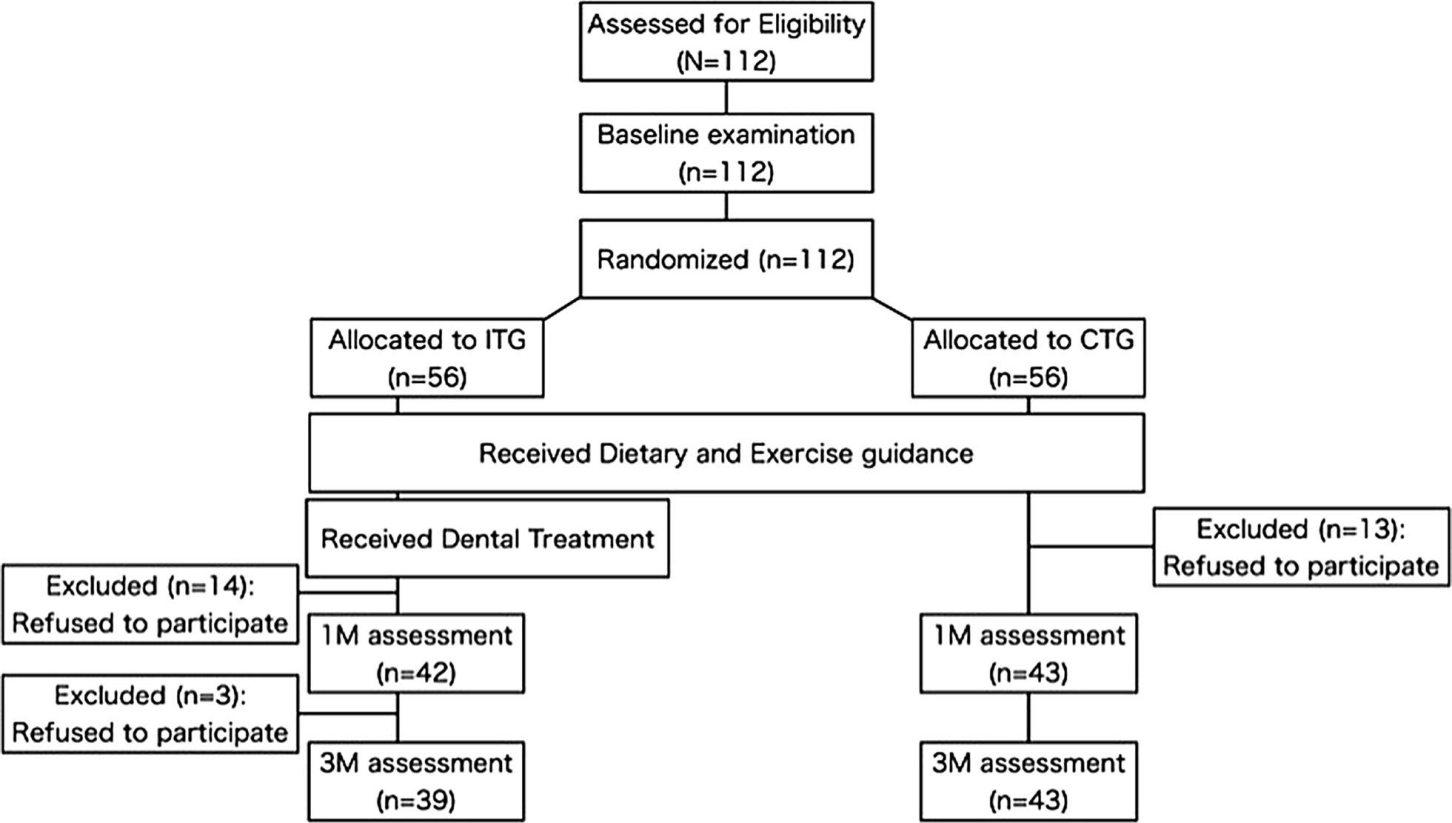
Flow chart of participants throughout the study

A total of 112 patients visited TMDU Dental Hospital to participate in this study. In the beginning, 100 participants were intended for this study; however, when the target number was recruited, there were 25 dropouts. Because the dropout rate was expected to be < 20%, additional recruitment was done. As shown in Fig. 1, 82 patients completed up to 3M, and 30 dropped out. Among those that completed the trial, 39 were assigned to ITG (men 26, women 13) and 43 to CTG (men 22, women 21). (Fig. 1) There was no significant difference in age, sex, waist circumference, weight, and BMI at base line between groups. All participants needed periodontal treatment. At the same time, one participant from ITG and two from CTG needed fixed partial dentures, and 12 participants from ITG and 14 from CTG needed removable partial dentures to replace the missing teeth (Table 1).

**Table 1:** Baseline characteristics of the participants

	Group		Total (n = 82)	
	Control (n = 43)	Intervention (n = 39)		*p* value
Age (years), mean (SD)	56.7 (10.3)	58.4 (9.4)	57.5 (9.9)	0.454^a^
Gender, n (%)				0.183^b^
Male	22 (51.2%)	26 (66.7%)	48 (58.5%)	
Female	21 (48.8%)	13 (33.3%)	34 (41.5%)	
Body height (cm), mean (SD)	163.8 (8.2)	166.7 (8.5)	165.2 (8.4)	0.119^a^
Body weight (kg), mean (SD)	74.0 (14.2)	74.3 (9.6)	74.1 (12.1)	0.890^a^
BMI, mean (SD)	27.5 (4.2)	26.8 (3.3)	27.1 (3.8)	0.407^a^
Waist circumference (cm), mean (SD)	98.4 (9.2)	97.8 (6)	98.1 (7.8)	0.727^a^
Types of treatment in need, n (%)				
Periodontal treatment	43 (100%)	39 (100%)	82 (100%)	
Fabrication of fixed partial denture	2 (4.7%)	1 (2.6%)	3 (3.7%)	
Fabrication of removable partial denture	14 (9.3%)	12 (30.8%)	26 (31.7%)	
PD ≥ 4 mm (% of sites), mean (SD)	15.1 (14.9)	19.1 (16.3)	17.0 (15.6)	0.243^a^
BOP (% of sites), mean (SD)	20.0 (18.4)	25.3 (21.4)	22.6 (19.9)	0.235^a^
Missing tooth (n), mean (SD)	3.6 (4.9)	3.2 (2.9)	3.4 (4.1)	0.658^a^

Data are presented as mean (standard deviation) (for all such values)

^a^Statistical analysis using student’s *t* test

^b^Statistical analysis using chi-square test

Results of comparisons between groups are shown in Table 2. Body water rate (*p* = 0.043) was significantly higher, while PD ≥ 4 mm (*p* = 0.000) and BOP (*p* = 0.001) were significantly lower in ITG than in CTG at 1M. Simultaneously, FBS level tended to be lower in ITG than in CTG. Lean mass (*p* = 0.037) and muscle mass (*p* = 0.035) were significantly higher, and body weight (*p* = 0.044), PD ≥ 4 mm (*p* = 0.000), and BOP (*p* = 0.002) were significantly lower in ITG than in CTG at 3M. Further, BMI tended to be lower in ITG than in CTG. Comparisons within groups, shown in Table 3, revealed anthropometric improvements in both groups at BL-1M and BL-3M. At BL-1M, ITG had marked significant increase in body water rate (*p* = 0.000) and significant decrease in body fat rate (*p* = 0.007), fat mass (*p* = 0.005), waist circumference (*p* = 0.004), systolic (*p* = 0.000) and diastolic (*p* = 0.004) blood pressures, PD ≥ 4 mm (*p* = 0.000) and BOP (*p* = 0.000). Along with that, body weight had tendency of decrement in ITG. Within CTG, significant reductions in waist circumference (*p* = 0.003), and systolic (*p* = 0.000) and diastolic (*p* = 0.016) blood pressures were observed. Moreover, at BL-3M, ITG had marked significant decrease in body weight (*p* = 0.014), BMI (*p* = 0.016), waist circumference (*p* = 0.000), systolic blood pressure (*p* = 0.001), PD ≥ 4 mm (*p* = 0.000), and BOP (*p* = 0.000). At the same time, tendency of decrease in fat mass and diastolic blood pressure was observed in ITG. Within CTG, significant reduction in waist circumference (*p* = 0.001), and systolic (*p* = 0.000) and diastolic blood pressures (*p* = 0.020) were marked. Furthermore, tendency of decrease in body fat rate, and tendency of increment in lean mass and muscle mass were observed in CTG. At 1M–3M, significant reductions in body water rate (*p* = 0.009) and tendency of increment in TG were marked in ITG.

**Table 2:** Comparisons of anthropometric measurements, dental measurements, blood pressure, and blood samples between the control group (n = 43) and the intervention group (n = 39) before and after treatment

	Baseline		1M			3M		
	Mean		Mean		*p* value*	Mean		*p* value*
	Control	Intervention	Control	Intervention		Control	Intervention	
Weight (kg)	74.0 (14.2)	74.3 (9.6)	73.8 (14.9)	73.5 (9.1)	0.17	73.9 (15.2)	73 (9.2)	0.044*
Body fat rate (%)	32.2 (10.3)	29.7 (8.1)	31.7 (9.8)	28.7 (8)	0.16	31.3 (10.4)	28.9 (7.9)	0.89
Body fat mass (kg)	24.3 (10.5)	22.1 (7)	23.9 (10.5)	21.1 (6.7)	0.12	23.7 (11)	21.3 (6.6)	0.45
Lean mass (kg)	49.7 (10.1)	52.2 (8.9)	49.9 (9.8)	52.4 (8.8)	0.76	50.3 (10.1)	51.9 (8.7)	0.037*
Muscle mass (kg)	47 (9.6)	49.4 (8.6)	47.1 (9.4)	49.6 (8.4)	0.76	47.5 (9.7)	49.1 (8.4)	0.035*
Estimated bone mass (kg)	2.7 (0.5)	2.8 (0.4)	2.8 (0.4)	2.8 (0.4)	0.8	2.8 (0.4)	2.8 (0.4)	0.11
Body water mass (kg)	35.8 (6.4)	36.1 (5.4)	35.8 (5.9)	36.7 (5.6)	0.18	36 (5.8)	36 (5.5)	0.24
Body water rate (%)	48.7 (5.8)	48.7 (4.2)	49 (5.3)	49.9 (4.3)	0.043*	49.3 (5.9)	49.4 (4.5)	0.77
BMI	27.5 (4.2)	26.8 (3.3)	27.4 (4.4)	26.5 (3.3)	0.22	27.5 (4.5)	26.3 (3.2)	0.052
Waist circumference (cm)	98.4 (9.2)	97.8 (6)	96.8 (9.1)	95.8 (6.5)	0.57	96.1 (9.4)	95.1 (6.2)	0.67
Systolic blood pressure (mmHg)	136.7 (17)	140.9 (16.4)	126.6 (13.9)	130.4 (13.3)	0.47	129 (14.5)	130.4 (13.9)	0.6
Diastolic blood pressure (mmHg)	80.8 (11.8)	79 (10.6)	76.8 (9.9)	74.5 (9.5)	0.54	77.1 (11.6)	75.9 (8.8)	0.84
LDL (mg/dl)	128.7 (38.3)	137.6 (35.2)	129 (31.3)	135.2 (36.9)	0.54	130 (26.2)	131.7 (37.3)	0.19
TG (mg/dl)	188.6 (383.9)	146.5 (58.5)	150.2 (100.4)	140.1 (70.3)	0.66	152.4 (127.5)	161.5 (77.9)	0.28
HDL (mg/dl)	64.9 (19.9)	58.9 (12.7)	63.5 (17.3)	60 (13.6)	0.2	65.6 (18.8)	59.4 (15.2)	0.94
FBS (mg/dl)	96.2 (11.4)	97.2 (24.9)	101.2 (17.2)	96.4 (16.2)	0.098	98.2 (21.2)	96.2 (16.1)	0.42
HbA1c (%) NGSP	5.6 (0.4)	5.6 (0.4)	5.6 (0.4)	5.5 (0.3)	0.18	5.6 (0.5)	5.53 (0.4)	0.28
PD ≥ 4 mm (% of sites)	15.1 (14.9)	19.1 (16.3)	15.8 (16.0)	11.3 (10.0)	0.000*	16.0 (16.5)	10.4 (10.2)	0.000*
BOP (% of sites)	20.0 (18.4)	25.3 (21.4)	19.4 (19.2)	14.3 (11.5)	0.001*	18.7 (18.7)	12.9 (12.9)	0.002*

Data are presented as mean (standard deviation) (for all such values). Statistical analysis using ANCOVA

*Statistically significant *p* values for between-group differences determined by ANCOVA with sex and baseline values as covariate

**Table 3:** Comparisons of changes in anthropometric measurements, dental measurements, blood pressure, and blood samples within the control (n = 43) and intervention (n = 39) groups before and after treatment

Time point	Control		Intervention	
	Difference in mean value	*p* value*	Difference in mean value	*p* value*
Weight (kg)				
Baseline-1 months	0.17	1.000	0.84	0.097
Baseline-3 months	0.05	1.000	1.35	0.014*
1–3 months	− 0.13	1.000	0.5	0.710
Body fat rate (%)				
Baseline-1 months	0.49	0.680	1.02	0.007*
Baseline-3 months	0.94	0.085	0.76	0.240
1–3 months	0.45	0.798	− 0.26	1.000
Body fat mass (kg)				
Baseline-1 months	0.34	1.000	1.01	0.005*
Baseline-3 months	0.58	0.711	0.97	0.055
1–3 months	0.24	1.000	− 0.04	1.000
Lean mass (kg)				
Baseline-1 months	− 0.16	1.000	− 0.17	1.000
Baseline-3 months	− 0.53	0.098	0.37	0.770
1–3 months	− 0.37	0.630	0.54	0.260
Muscle mass (kg)				
Baseline-1 months	− 0.14	1.000	− 0.15	1.000
Baseline-3 months	− 0.51	0.096	0.35	0.760
1–3 months	− 0.37	0.560	0.50	0.260
Estimated bone mass (kg)				
Baseline-1 months	− 0.02	0.830	− 0.02	0.680
Baseline-3 months	− 0.06	0.230	0.02	1.000
1–3 months	− 0.01	1.000	0.04	0.300
Body water mass (kg)				
Baseline-1 months	0.00	1.000	− 0.54	0.150
Baseline-3 months	− 0.21	1.000	0.11	1.000
1–3 months	− 0.22	0.750	0.654	0.009*
Body water rate (%)				
Baseline-1 months	− 0.24	1.000	− 1.24	0.000*
Baseline-3 months	− 0.56	0.550	− 0.74	0.059
1–3 months	− 3.26	0.670	0.51	0.210
BMI				
Baseline-1 months	0.05	1.000	0.28	0.130
Baseline-3 months	0.01	1.000	0.48	0.016*
1–3 months	− 0.04	1.000	0.20	0.650
Waist circumference (cm)				
Baseline-1 months	1.60	0.003*	1.92	0.004*
Baseline-3 months	2.27	0.001*	2.67	0.000*
1–3 months	0.67	0.250	0.75	0.390
Systolic blood pressure (mmHg)				
Baseline-1 months	10.09	0.000*	10.51	0.000*
Baseline-3 months	7.72	0.000*	10.49	0.001*
1–3 months	− 2.37	0.750	− 0.018	0.750
Diastolic blood pressure (mmHg)				
Baseline-1 months	4.07	0.016*	4.54	0.004*
Baseline-3 months	3.73	0.020*	3.12	0.073
1–3 months	− 0.34	1.000	− 1.42	1.000
LDL (mg/dl)				
Baseline-1 months	− 0.23	1.000	2.33	1.000
Baseline-3 months	− 1.21	1.000	5.85	0.410
1–3 months	− 0.98	1.000	3.51	1.000
TG (mg/dl)				
Baseline-1 months	38.42	1.000	6.46	1.000
Baseline-3 months	36.19	1.000	− 14.95	0.720
1–3 months	− 2.23	1.000	− 21.41	0.091
HDL (mg/dl)				
Baseline-1 months	1.42	0.650	− 1.10	1.000
Baseline-3 months	− 0.67	1.000	− 0.46	1.000
1–3 months	− 2.09	0.370	0.64	1.000
FBS (mg/dl)				
Baseline-1 months	− 5.05	0.170	0.85	1.000
Baseline-3 months	− 2.05	1.000	1.08	1.000
1–3 months	3.00	1.000	0.23	1.000
HbA1c (%) NGSP				
Baseline-1 months	0.03	0.850	0.06	0.300
Baseline-3 months	0.01	1.000	0.04	0.750
1–3 months	− 0.02	1.000	− 0.02	1.000
PD ≥ 4 mm (% of sites)				
Baseline-1 months	− 0.70	1.000	7.80	0.000
Baseline-3 months	− 0.90	1.000	8.70	0.000
1–3 months	− 0.20	1.000	0.90	1.000
BOP (% of sites)				
Baseline-1 months	0.60	1.000	11.00	0.000
Baseline-3 months	1.30	1.000	12.40	0.000
1–3 months	0.70	1.000	1.40	0.949

Data were presented as differences in mean value. A negative value for within-group changes denotes an increase of variable between time points. Statistical analysis using Repeated ANOVA

*Statistically significant *p* values for within-group changes

Subgroup analysis, shown in Tables 4 and 5, was performed to assess the deviation from treatment types. Prevalence of periodontitis was 100% among all 82 participants. Among them, 29 (ITG: CTG = 13:16) participants had missing teeth, and 53 (ITG: CTG = 26:27) had periodontitis alone. Among the participants with periodontitis alone, body fat mass (*p* = 0.004) was significantly higher, and body fat rate (*p* = 0.005) and body water rate (*p* = 0.048) were significantly lower in ITG than in CTG at 1M. Among participants with missing teeth, systolic blood pressure (*p* = 0.020) was significantly higher at 1M in ITG than in CTG. At 3M, lean mass (*p* = 0.005), muscle mass (*p* = 0.005), estimated bone mass (*p* = 0.009), and body water mass (*p* = 0.017) were significantly lower in ITG than in CTG.

**Table 4:** Comparisons of anthropometric measurements, dental measurements, blood pressure, and blood samples between the control group (n = 16) and the intervention group (n = 13) before and after treatment among patients with missing teeth

	Baseline		1M			3M		
	Mean		Mean		*p* value*	Mean		*p* value*
	Control	Intervention	Control	Intervention		Control	Intervention	
Weight (kg)	79.7 (18.3)	71.1 (9.1)	79.9 (19.5)	71.3 (8.5)	0.969	80.4 (20.1)	69.3 (8.5)	0.167
Body fat rate (%)	35.8 (9.6)	27.9 (6.7)	35.0 (9.1)	27.6 (6.1)	0.691	34.2 (10.5)	27.8 (5.8)	0.566
Body fat mass (kg)	29.1 (11.9)	19.9 (5.4)	28.7 (12.3)	19.7 (4.7)	0.710	28.4 (13.3)	19.3 (4.7)	0.984
Lean mass (kg)	50.6 (11.8)	51.2 (8.1)	51.2 (11.5)	51.6 (7.6)	0.928	52.0 (12.1)	50.0 (7.1)	0.005*
Muscle mass (kg)	47.8 (11.3)	48.5 (7.8)	48.3 (11.0)	48.8 (7.3)	0.918	49.1 (11.6)	47.3 (6.9)	0.005*
Estimated bone mass (kg)	2.8 (0.5)	2.8 (0.4)	2.9 (0.5)	2.8 (0.4)	0.962	2.9 (0.5)	2.7 (0.3)	0.009*
Body water mass (kg)	36.7 (7.7)	35.3 (5.1)	36.8 (7.2)	36.2 (4.6)	0.789	37.1 (6.6)	34.9 (4.6)	0.017*
Body water rate (%)	46.5 (5.4)	49.8 (3.9)	46.8 (5.4)	50.8 (2.7)	0.168	47.2 (6.3)	50.4 (4.1)	0.799
BMI	29.1 (5.0)	26.4 (2.8)	29.2 (5.5)	26.6 (2.8)	0.944	29.3 (5.5)	25.8 (2.6)	0.111
Waist circumference (cm)	103.3 (11.1)	97.2 (4.9)	102.3 (10.5)	95.3 (7.0)	0.380	101.8 (10.7)	93.6 (5.2)	0.094
Systolic blood pressure (mmHg)	136.5 (18.5)	140.5 (15.4)	122.4 (10.7)	133.1 (14.5)	0.020*	130.4 (12.4)	126.0 (10.5)	0.061
Diastolic blood pressure (mmHg)	77.6 (10.7)	77.6 (7.1)	73.3 (8.5)	73.0 (8.8)	0.926	76.9 (12.3)	72.8 (6.5)	0.137
LDL (mg/dl)	129.7 (23.9)	132.2 (37.1)	126.7 (26.9)	126.5 (42.0)	0.449	133.2 (26.0)	134.4 (44.2)	0.657
TG (mg/dl)	155.5 (113.4)	132.6 (42.3)	157.3 (80.8)	135.0 (58.3)	0.498	152.0 (111.6)	153.8 (61.0)	0.691
HDL (mg/dl)	60.1 (13.8)	57.2 (10.3)	58.2 (12.7)	57.2 (10.2)	0.603	62.1 (14.3)	55.1 (11.9)	0.238
FBS (mg/dl)	99.7 (11.8)	105.2 (36.5)	105.6 (21.3)	99.5 (20.7)	0.171	105.4 (28.3)	98.8 (14.8)	0.170
HbA1c (%) NGSP	5.8 (0.5)	5.7 (0.4)	5.7 (0.5)	5.7 (0.4)	0.444	5.8 (0.5)	5.6 (0.4)	0.454
PD ≥ 4 mm (% of sites)	17.6 (15.6)	23.7 (17.7)	17.2 (16.5)	12.9 (9.9)	0.006*	17.1 (15.5)	11.8 (10.3)	0.004*
BOP (% of sites)	25.1 (22.3)	31.3 (20.3)	21.4 (20.4)	18.0 (13.0)	0.130	20.5 (19.2)	15.8 (13.4)	0.129

Data are presented as mean (standard deviation) (for all such values). Statistical analysis using ANCOVA

*Statistically significant *p* values for between-group differences determined by ANCOVA with sex and baseline values as covariate

**Table 5:** Comparisons of anthropometric measurements, dental measurements, blood pressure, and blood samples between the control group (n = 27) and the intervention group (n = 26) before and after treatment among patients with periodontitis alone

	Baseline		1M			3M		
	Mean		Mean		*p* value*	Mean		*p* value*
	Control	Intervention	Control	Intervention		Control	Intervention	
Weight (kg)	70.6 (10.0)	76.9.50	70.2 (10.1)	74.6 (9.4)	0.089	70.1 (10.0)	74.8 (9.1)	0.495
Body fat rate (%)	30.0 (10.3)	30.6 (8.7)	29.8 (10.0)	29.2 (8.8)	0.005*	29.5 (10.1)	29.5 (8.8)	0.280
Body fat mass (kg)	21.4 (8.5)	23.3 (7.6)	21.1 (8.4)	21.8 (7.4)	0.004*	20.9 (8.5)	22.1 (7.3)	0.259
Lean mass (kg)	49.2 (9.1)	52.7 (9.4)	49.1 (8.7)	52.8 (9.4)	0.444	49.2 (8.8)	52.8 (9.4)	0.679
Muscle mass (kg)	46.5 (8.7)	49.9 (9.0)	46.4 (8.4)	49.9 (9.1)	0.461	46.5 (8.5)	49.9 (9.1)	0.723
Estimated bone mass (kg)	2.7 (0.4)	2.8 (0.4)	2.7 (0.4)	2.9 (0.4)	0.263	2.7 (0.4)	2.9 (0.4)	0.225
Body water mass (kg)	35.2 (5.7)	36.5 (5.6)	35.1 (5.1)	36.9 (6.1)	0.210	35.3 (5.3)	36.6 (5.9)	0.931
Body water rate (%)	50.1 (5.7)	48.1 (4.3)	50.3 (5.0)	49.5 (4.9)	0.048*	50.6 (5.4)	48.9 (4.7)	0.905
BMI	26.5 (3.3)	26.9 (3.6)	26.4 (3.4)	26.4 (3.6)	0.056	26.3 (3.3)	26.5 (3.5)	0.371
Waist circumference (cm)	95.4 (6.4)	98.0 (6.6)	93.5 (6.3)	96.1 (6.4)	0.698	92.7 (6.6)	95.8 (606)	0.292
Systolic blood pressure (mmHg)	136.8 (16.5)	141.0 (17.2)	129.1 (15.1)	129.0 (12.7)	0.497	128.1 (15.7)	132.6 (15.1)	0.617
Diastolic blood pressure (mmHg)	82.7 (12.2)	79.7 (12.1)	78.8 (10.3)	75.1 (10.0)	0.408	77.2 (11.4)	77.4 (9.6)	0.450
LDL (mg/dl)	128.2 (31.1)	140.2 (34.6)	130.3 (34.0)	139.6 (34.2)	0.813	128.0 (26.6)	130.4 (34.2)	0.202
TG (mg/dl)	208.2 (479.2)	153.5 (64.7)	145.9 (111.6)	142.6 (76.5)	0.928	152.6 (138.2)	165.3 (85.9)	0.183
HDL (mg/dl)	67.8 (21.1)	59.8 (13.9)	66.7 (19.0)	61.4 (15.0)	0.308	67.7 (21.0)	61.5 (16.5)	0.517
FBS (mg/dl)	94.1 (10.8)	93.3 (15.8)	98.6 (14.1)	94.8 (13.6)	0.323	94.0 (14.7)	94.8 (16.8)	0.758
HbA1c (%) NGSP	5.6 (0.3)	5.5 (0.4)	5.6 (0.4)	5.5 (0.3)	0.068	5.5 (0.4)	5.5 (0.3)	0.520
PD ≥ 4 mm (% of sites)	13.6 (14.5)	16.8 (15.4)	14.9 (15.9)	10.5 (10.1)	0.001*	15.4 (17.3)	9.7 (10.3)	0.000*
BOP (% of sites)	17.1 (15.3)	22.3 (21.7)	18.2 (18.7)	12.5 (10.4)	0.006*	17.7 (18.7)	11.4 (12.7)	0.006*

Data are presented as mean (standard deviation) (for all such values). Statistical analysis using ANCOVA

*Statistically significant *p* values for between-group differences determined by ANCOVA with sex and baseline values as covariate

Results of comparison between dropouts and those who retained through out the study at baseline is shown in Table 6. There were significant differences in height and missing teeth at baseline between participants who retained throughout the study period and those who retired. Height (*p* = 0.023) and missing teeth (*p* = 0.014) were significantly higher among the dropouts, respectively. Periodontal status (PPD ≥ 4 mm and BOP) among dropouts tended to be worse than those who retained.

**Table 6:** Baseline characteristics of the Dropout group and the difference with the Retained group

	Dropout group (D)			Retained group (R)	Difference between D–R
	Group		Total (n = 30)	Total (n = 82)	
	Control (n = 13)	Intervention (n = 17)			*p* value
Age (years), mean (SD)	55.8 (11.8)		54.7 (10.3)	57.5 (9.9)	0.201^a^
Gender, n (%)					0.269^b^
Male	11 (84.6%)	10 (58.8%)	21 (70%)	48 (58.5%)	
Female	2 (15.4%)	7 (41.2%)	9 (30%)	34 (41.5%)	
Body height (cm), mean (SD)	170.9 (7.7)	168 (7.2)	169.2 (7.4)	165.2 (8.4)	0.023^a^*
Body weight (kg), mean (SD)	77.4 (10.2)	76.9 (14.5)	77.1 (12.6)	74.1 (12.1)	0.260^a^
BMI, mean (SD)	26.6 (3.4)	27.1 (4.0)	26.9 (3.7)	27.1 (3.8)	0.743^a^
Waist circumference (cm), mean (SD)	91.7 (8.0)	98.9 (8.9)	98.4 (8.4)	98.1 (7.8)	0.851^a^
Types of treatment in need, n (%)					
Periodontal treatment	13(100%)	17 (100%)	30 (100%)	82 (100%)	
Fabrication of fixed partial denture	1 (7.7%)	1 (5.9%)	2 (6.7%)	3 (3.7%)	
Fabrication of removable partial denture	4 (30.8%)	9 (52.9%)	13 (43.3%)	26 (31.7%)	
PD ≥ 4 mm (% of sites), mean (SD)	28.8 (23.1)	19.5 (16.4)	23.6 (19.8)	17.0 (15.6)	0.070^a^
BOP (% of sites), mean (SD)	30.6 (20.9)	29.6 (23.7)	30.0 (22.1)	22.6 (19.9)	0.092^a^
Missing tooth (n), mean (SD)	4.9 (3.9)	6.2 (5.3)	5.7 (4.7)	3.4 (4.1)	0.014^a^*

Data are presented as mean (standard deviation) (for all such values)

^a^Statistical analysis using student’s t-test

^b^Statistical analysis using chi-square test

*Statistically significant *p* values for between-group differences

## Discussion

This trial was a novel approach to identify the combined effect of dental intervention, including periodontal treatment and prosthodontics, with lifestyle guidance on MetS. The null hypothesis was rejected because body water rate was significantly higher, and FBS level tended to be lower in ITG than in CTG at 1M. In addition, significantly higher lean mass and muscle mass, significantly lower body weight, and tendency of lower BMI in ITG than in CTG at 3M were observed.

Higher body water rate, observed in ITG, implied increase in body tissue other than fat. The changes in body water, weight, lean mass, and muscle mass indicated reduction of weight without losing the essential components that build the body. Moreover, along with the reduction of weight, increase in the proportion of muscle was observed in the ITG. Difference in body composition between ITG and CTG could be considered as an effect of the dental treatment that had been performed in ITG alone. Thus, enhancement in self-awareness and suppression of inflammatory cascade could be the possible causes. Kobayashi [13] and Ylöstalo [31] discovered that frequency of oral health behaviors, which is considered as the ability of self-awareness, is related to lower prevalence of MetS or obesity. Maintaining the oral environment by TBI and periodontal treatment might have motivated participants’ self-awareness, which could have encouraged their improvement in diet and exercise. Moreover, Wilcox [32] and Bullon [33] investigated the inflammatory cascade between periodontitis and MetS. IR, strongly related to diabetes, increases with the existence of excessive visceral fat and lack of exercise. Adipocytokine, which is secreted from visceral fat, mediates the release of inflammatory cytokines and reactive oxygen species (ROS) from leucocytes that would further activate host immune reaction, resulting in periodontium destruction. Dandona [34] suggested that consistent periodontitis leads to further production of inflammatory cytokines, which brings about systemic inflammation and increase in IR, leading to progression of diabetes. Furthermore, ROS and *Porphyromonas gingivalis* have also been causes of vascular dysfunction, leading to hypertension and atherosclerosis [35]. In this study, it is considered that dental treatment could block the cascade by maintaining a good standard of oral hygiene and relieving inflammation, which may lead to possible improvements in MetS and its related factors.

Considering the results of comparisons within groups, waist circumference in both groups declined, implying a reduction in visceral fat in both groups. According to Fang, change in waist circumference is caused by fluctuation of the visceral fat [36]. Comparing current results with this finding, dietary and exercise guidance might have encouraged reduction of caloric intake and increase of metabolism due to exercise, which may have led to reduction of visceral fat. Moreover, FBS tendency to be lower at 1M in ITG than in CTG could be attributed to this cascade. However, the decrement of blood pressure and waist circumference in both groups seemed to be obtained from dietary and exercise guidance rather than dental treatment.

Body water mass LDL, HDL, and HbA1c changes had no marked significance or tendency in the overall result. Change of body water may not have been detected by mass because the variation of total weight between individuals was sizeable. Regarding LDL results, our result seemed to be consistent with that in a previous report by Tuomilehto [37] and Munakata [29], in which it was found that LDL level had not changed after significant reduction of weight among obese participants. Moreover, Siri-Tarino and Krauss explained that food intake would not be directly reflected on blood test data because the lipoprotein particles need to be produced in the liver before they are distributed into peripheral blood as HDL and LDL [38]. If long-term outcome measurement could be performed, the result might have become more obvious. However, the intake of saturated fatty acid should be assessed later on to investigate the dietary effect. According to Little, HbA1c has been used as an indication of average glucose level because it reflects blood glucose of the past 8–12 weeks [39]. In addition, the intra-individual variation in the non-diabetic population is very small. Considering the study period and diabetic statuses of the participants in this study, the result seems to be reasonable.

Subgroup analysis was performed to assess the deviation from treatment types. Comparing subgroup results with the overall results of the study, significant deviation has not been observed in subgroups. Both subgroups showed similar trends with the overall result, although the missing teeth group appeared to lack in sample size. At baseline, height and weight were significantly higher in ITG than in CTG among patients with periodontitis alone. However, body fat rate and body fat mass were lower in ITG than in CTG among patients with missing teeth at baseline. Considering these deviations in baseline data, there seems to be no significant deviation between subgroups.

Munakata performed an RCT, intending to reduce the weights of patients with MetS by lifestyle guidance [29]. Patients with MetS were provided initial lifestyle guidance. Thereafter, the experimental group received multiple individualized lectures every 2 months, while the control group was requested to reduce weight on their own. Improvements on waist circumference and FBS level in the experimental group compared with the control group were revealed. Waist circumference and FBS level in our study did not show significant improvement in comparison between groups. Compared with our intervention, their lifestyle guidance had been remarkably intense, and this intensity may have taken effect. Baeza had compared nine RCTs that examined the effect of dental treatment on patients with diabetes, and they revealed SRP to be effective in reducing HbA1c levels [14]. Periodontal intervention included TBI and SRP with the primary outcome on HbA1c and CRP levels. Saengtipbovorn and Taneepanichskul had provided lifestyle guidance followed by oral hygiene instructions to the experimental group and medical consultation to the control group among patients with diabetes in their RCT [18]. As a result, HbA1c and FBS levels, and periodontal status improved 6 months after intervention. Compared with these two trials, our participants were mainly those who had abdominal obesity; therefore, the intra-individual variation in HbA1c level was considered very small. Having a tendency of lower FBS level in ITG than in CTG, periodontal treatment had possibly improved diabetic statuses of our participants in ITG. Papageorgiou compared 15 RCTs that had examined the effect of periodontal treatment on obese participants [16]. TNFα and HbA1c levels after periodontal therapy were significantly higher in overweight/obese patients than in normal weight patients. Lopez reported that reduction of periodontal inflammation either with SRP and systemic antibiotics with TBI significantly reduced CRP levels at 9 months in patients with MetS, after intervention [17]. The difference between our trial and these two trials was the existence of lifestyle guidance. Anthropometric improvement in the intervention group of our study may be due to the combination of dental intervention and lifestyle guidance. Improvement in glycemic condition seemed to be the common point with Lopez’ study.

### Limitation

Below are the possible limitations of this study. First, we guided the participants on diet and exercise. Participants were required to respond to the brief diet-history questionnaire (BDHQ); however, this would not be assessed in this paper. However, assessment for exercise intensity was not performed in the first place. Second, the final assessment of the outcomes was at post 3 months in this study because 3 months appeared to be the ethical limit to keep patients from needed dental treatment. If we could come out with a good solution for this point, long-term observation would be desirable. Third, this trial was voluntary; the participants were motivated to get healthier and to undergo dental treatment for dental health. Therefore, it seems to be doubtful whether the outcomes were simply from the intervention or from their motivations. Fourth, setting waist circumference as a primary outcome could be considered as one of the limitations. To assess the visceral fat mass more accurately, computed tomography (CT) or magnetic resonance imaging (MRI) scans would be the most appropriate procedures [36]. Although CT has a risk of radiation exposure, requiring CT and MRI scans for all participants in each assessment (BL, 1M, and 3M) would also incur considerable cost and time. According to Fang, visceral fat mass on CT has been correlated with waist circumference [36]. From the report of Matsuzawa, waist circumferences of 85 cm (men) and 90 cm (women) are equivalent to 100 cm^2^ of visceral fat area, and waist circumference is more closely related to visceral fat than BMI [26]. Considering the results of these studies and the feasibility of the procedure, we determined to use waist circumference as the primary outcome to assess the effect of the intervention. Fifth, at baseline, height and missing teeth were significantly higher, and periodontal status (PPD ≥ 4 mm and BOP) tended to be worse among the dropouts than those who retained. We have done student’s t-test to examine if there is any significant difference between controls and treatment group of the dropouts, in which we found that there was no significant difference between each group. It remains unclear, whether the dropouts had any bias effect towards the result of this study, but regarding the result from between-group (Treatment–Control) difference in dropouts, we consider that losing those participants had little effect.

For the future perspective, changes in participants’ dietary patterns would be clarified by analyzing the results of BDHQs, which may deepen our understanding regarding the results obtained in this study. If another project were to be launched, a study design that enables long-term observation would be desired to assess the longevity of the treatment effect and the rebound effect. Evaluation of exercise intensity, which was not applied in this project, would also enable multilateral analysis on the effect of lifestyle guidance.

## Conclusion

As a conclusion, the combination of dental intervention and lifestyle guidance improved both anthropometric and periodontal statuses of patients with MetS.

## Data Availability

The datasets used and/or analyzed during the current study available from the corresponding author on reasonable request.
